# Editorial: Beyond the borders: The gates and fences of neuroimmune interaction, volume II

**DOI:** 10.3389/fnint.2022.968249

**Published:** 2022-08-02

**Authors:** Beatriz Gómez-González, Gabriela Hurtado-Alvarado, Javier Velázquez-Moctezuma

**Affiliations:** ^1^Neuroscience Area, Department of Biology of Reproduction, Universidad Autónoma Metropolitana, Unidad Iztapalapa, Mexico City, Mexico; ^2^Instituto de Investigaciones Biomédicas, Universidad Nacional Autónoma de México, Ciudad Universitaria, Mexico City, Mexico

**Keywords:** neuroimmune, neuroendocrine, obesity, cognitive deficit, inflammation, microbioma

The classic compartmentalization in the study of the immune and the central nervous system has been met with overwhelming results that show there is a close relationship between the brain and the immune system. Early data on this subject has revealed that emotions play a major role in the development of some diseases in which the immune system is involved (Rasmussen, 1969). Early observations on the impact of stress on the immune response or the conditioned stimuli modulating the immune system further support this notion (Solomon et al., 1974; Ader and Cohen, 1975). Recently, it has been acknowledged that the reciprocal interaction between the neural and immune systems plays a key role in the development of autoimmune diseases (Sharif et al., 2018; Ilchmann-Diounou and Menard, 2020) and cancer (Antoni and Dhabhar, 2019; Chang et al., 2022).

Anatomical and biochemical evidence in recent decades strongly confirms the close relationship between the nervous and immune systems (Besedovsky and Rey, 2007; Huh and Veiga-Fernandes, 2020). Indeed, reciprocal neuro-immune interaction has been extended to also include the endocrine system, therefore constituting the neuro-immune-endocrine system. With this new perspective in mind, physiopathological manifestations can no longer be analyzed as isolated phenomena. It is well-known that at the local level, the nervous system produces immune factors, and the immune system produces neuroendocrine mediators (Besedovsky and Rey, 2007). The innervation of immune organs by the peripheral nerve endings results in the fine modulation of the immune response (Huh and Veiga-Fernandes, 2020). To complete the circle, immune mediators also play an important role in neural physiology, not only regulating neural excitability but also influencing and shaping synaptic plasticity (Levin and Godukhin, 2017). Considering the neuro-immune-endocrine interplay, several pathologies located mainly in the nervous system, in the endocrine system, or as part of the immune response are now analyzed as organic disturbances that include the reciprocal interactions between the three systems (Sharif et al., 2018; Antoni and Dhabhar, 2019; Ilchmann-Diounou and Menard, 2020; Chang et al., 2022).

The present volume includes novel studies that reflect this vision extensively. Samano et al. describe recent advances in the discovery of the role of circRNAs on spinal cord injury, with special emphasis on their modulation of neuroinflammation, proposing their potential use as biomarkers or therapeutic targets. Corona et al. review prolactin serum changes in chronic kidney disease and suggest that hyperprolactinemia exacerbates peripheral inflammation, orchestrating central and peripheral changes, which lead to olfactory impairment and malnutrition. García-Aviles et al. propose a hypothesis regarding the mechanism by which sleep loss induces cognitive deficits, suggesting a key role for altered insulin signaling in mediating the hippocampal physiological changes underlying cognitive impairment, positioning cytokines as intermediators. Salas-Venegas et al. extensively review the evidence of peripheral and central inflammation in obesity and its relationship with the blood-brain barrier dysfunction and cognitive deficit; emphasizing the role of current and new therapies in modulating inflammation and therefore preventing obesity-related cognitive deficits. Finally, based on the importance of microbiota in normal brain physiology, Pacheco-Lopez et al. provide a theoretical framework for future research in the field of social microbioma, suggesting a co-evolutionary model of social complexity, cerebral cortex evolution, and microbiota diversity.

From now on, the most common human pathologies, including cancer or infectious diseases, should be treated by always taking the fact that they are not a local or particular disturbance into consideration, as they can reflect a misbalance of the interactions of the nervous, the immune, and the endocrine systems. This integral framework opens an innovative vision for the discovery and use of modern treatments for human diseases. This new perspective on human pathology encourages and promotes interdisciplinary collaboration to prevent and treat diseases, and, finally prevent and promote the absence of illness and the wellbeing of humankind.

## Author contributions

BG-G wrote the manuscript, edited, and reviewed the final version of the manuscript. JV-M wrote the manuscript and approved the final version. GH-A reviewed and approved the final version of the manuscript. All authors contributed to the article and approved the submitted version.

## Conflict of interest

The authors declare that the research was conducted in the absence of any commercial or financial relationships that could be construed as a potential conflict of interest.

## Publisher's note

All claims expressed in this article are solely those of the authors and do not necessarily represent those of their affiliated organizations, or those of the publisher, the editors and the reviewers. Any product that may be evaluated in this article, or claim that may be made by its manufacturer, is not guaranteed or endorsed by the publisher.
